# Bioinformatics and High-Performance Computing Methods for Deciphering and Fighting COVID-19—Editorial

**DOI:** 10.3390/biotech11040047

**Published:** 2022-10-15

**Authors:** Mario Cannataro, Giuseppe Agapito

**Affiliations:** 1 Data Analytics Research Center, Department of Medical, Surgical Science, University “Magna Graecia” of Catanzaro, Catanzaro 88100, Italy; 2 Department of Legal, Historical, Economic and Social Sciences, University “Magna Graecia” of Catanzaro, Catanzaro 88100, Italy

The COVID-19 disease (Coronavirus Disease 19), caused by the SARS-CoV-2 virus (Severe Acute Respiratory Syndrome Coronavirus 2), has posed many challenges worldwide at various levels, with special focus to the biological, medical, and epidemiological ones. Since the appearance of the disease at the end of 2019, an unprecedented effort in basic research, public health, epidemiology and control of virus spread has interested thousands of researchers and health professionals, with a huge production of scientific publications, drugs, vaccines and computer-based systems created to control the pandemics [1].

Several projects are attempting to gain knowledge by analyzing large-scale clinical data about COVID-19 patients. Among the others, the Consortium for Clinical Characterization of COVID-19 by EHR (4CE), is an international effort for studying the COVID-19 pandemic by integrating and analyzing clinical data about COVID-19 patients stored into Electronic Health Records (EHRs) belonging to hundred of hospitals located worldwide (https://covidclinical.net/, accessed on 12 October 2022). The Consortium includes more than 342 hospitals and six countries, and its main goal is to inform doctors, epidemiologists and the public about COVID-19 patients with data acquired through the health care process [2,3].

The main fields of research include: investigating the molecular basis of the disease, tracing virus mutations, developing SARS-CoV-2 genomes and variants databases, studying host–virus interactions, development of novel vaccines and drugs, adaptation of old drugs to the new disease (drug repurposing), collection and analysis of epidemiological data, analysis of electronic health records (EHRs) on a large scale, testing and tracing of people, infectious disease modeling, impact of COVID-19 on public health, effects of the pandemic at the emotional and behavioral level, etc. Each of the previous challenges has gained benefits from the solutions developed in several branches of computer science, with special focus on bioinformatics pipelines, biological databases, network analysis, and data and text mining [4,5,6,7,8].

This Special Issue presents several bioinformatics and computing methods addressing some of the previous challenges that are central for deciphering and for fighting the COVID-19 pandemic. In particular, it collects five research articles summarized below that touch on important topics such as: (i) software pipelines for analyzing SARS-CoV-2 viral sequences; (ii) databases for collecting and storing SARS-CoV-2 variants; (iii) an extended SIRD (Susceptible-Infected-Recovered-Deceased) model that has been used to characterize the Omicron wave in Brazil, South Africa, and Germany; (iv) a methodology for visualizing the temporal evolution of COVID-19 data and its application to evaluate the behavior of Italian regions during the first waves of viral infections; and finally; (v) a text-mining system that has been used for the characterization of writings by PASC (Post-acute Sequelae of COVID-19) patients as opposed to writings by health professionals and general reflections on COVID-19.

In VirusLab: A Tool for Customized SARS-CoV-2 Data Analysis [9], Pietro Pinoli, Anna Bernasconi, Anna Sandionigi and Stefano Ceri presented VirusLab, a software system for analyzing SARS-CoV-2 viral sequences and relating them to clinical information about the host. The system exploits ViruSurf, a database of public SARS-CoV-2 sequences, and VirusViz, a tool for visual analysis of search results. Authors described both the VirusLab architecture and how it can be used by the final users through a simple workflow. Among the provide functions, the *population report* provides a summary of the characteristics of a group of sequences, the *sequence data analysis* provides a bar plot representing the distribution of variants, and the *comparative data analysis* allows us to visually compare the variants distributions of multiple sub-populations. Authors foresee that VirusLab can support many research and therapeutic tasks within hospitals, or the tracing of viral sequences in viral surveillance.

In High Performance Integration Pipeline for Viral and Epitope Sequences [10], Tommaso Alfonsi, Pietro Pinoli and Arif Canakoglu presented an integrated pipeline to collect, transform, and integrate viral sequences of SARS-CoV-2, MERS, SARS-CoV, Ebola, and Dengue from four major database institutions (NCBI, COG-UK, GISAID, and NMDC). This pipeline allowed the development of VirusViz and EpiSurf, two data exploration interfaces, and of ViruSurf, one of the largest databases of viral sequences. The article is mainly devoted to describe the data model, the implementation and the performance evaluation of such database that, through a refinement process, was made more efficient, scalable, and general with respect to early versions. At the date of the article’s submission (March 2022), the database contains about 9.1 million SARS-CoV-2 sequences, and is a valuable tool for researchers interested in understanding the biological mechanisms of the viral infection. In addition, it is central in many analytic and visualization tools, such as ViruSurf, EpiSurf, VirusViz, and VirusLab, provided by the same research group.

In Characterisation of Omicron Variant during COVID-19 Pandemic and the Impact of Vaccination, Transmission Rate, Mortality, and Reinfection in South Africa, Germany, and Brazil [11], Carolina Ribeiro Xavier, Rafael Sachetto Oliveira, Vinícius da Fonseca Vieira, Marcelo Lobosco and Rodrigo Weber dos Santos employed and an extended version of an SIRD model able to take into account the effects of vaccination, time-dependent transmissibility rates, mortality, and potential reinfections. Authors modeled the Omicron wave in Brazil, South Africa, and Germany, showing that during Omicron, the transmissibility increased by five for Brazil and Germany and eight for South Africa, while the mortality was reduced by three-fold. Moreover, they also estimated reinfection in South Africa (that was 40% with only 29% of its population fully vaccinated), and in Brazil (that was 13% with over 70% and 80% of its population fully vaccinated and with at least one dose, respectively). The proposed models seem to be valuable tools for quantifying the impact of protocols and decisions in different populations.

In Application of CCTV Methodology to Analyze COVID-19 Evolution in Italy [12], Marianna Milano, Giuseppe Agapito and Mario Cannataro presented the COVID-19 Community Temporal Visualizer (CCTV) methodology and applied it to visualize COVID-19 data represented as graphs and to show their evolution in Italy along the various waves in 2020, 2021, and early 2022. Authors evaluated how Italy reacted to the pandemic in the first two waves of COVID-19, in which only containment measures were adopted, and after the start of the vaccination campaign. CCTV methodology allowed us to map the similarities in the behavior of Italian regions on a graph and to find how regions formed communities along time.

In Investigating Topic Modeling Techniques to Extract Meaningful Insights in Italian Long COVID Narration [5], Ileana Scarpino, Chiara Zucco, Rosarina Vallelunga, Francesco Luzza and Mario Cannataro applied different topic modeling techniques, such as Latent Dirichlet Allocation (LDA) and Bidirectional Encoder Representations (BERT) transformers, to analyze Narrative Medicine (NM) texts written by COVID-19 patients showing Post-acute Sequelae of COVID-19 (PASC) symptoms, as opposed to writings by health professionals and general reflections on COVID-19. The authors showed that the BERTopic-based approach outperforms the LDA-based approach by grouping in the same cluster the 97.26% of analyzed documents, and reaching an overall accuracy of 91.97%.

## Abbreviations

The following abbreviations are used in this manuscript:

SARS-CoV-2	Severe Acute Respiratory Syndrome Coronavirus 2
COVID-19	Coronavirus Disease 19
SIRD	Susceptible-Infected-Recovered-Deceased
EHR	Electronic Health Record
CCTV	COVID-19 Community Temporal Visualizer
LDA	Latent Dirichlet Allocation
BERT	Bidirectional Encoder Representations
NM	Narrative Medicine
PASC	Post-acute Sequelae of COVID-19
